# Evaluation of toxicity, local biocompatibility, biodegradation, and systemic metabolism of cellulose/alginate/strontium apatite membranes implanted subcutaneously in mice

**DOI:** 10.1590/acb401925

**Published:** 2025-03-10

**Authors:** Juliana Dantas da Costa, Erika Iara de Souza Araújo, Juan Feliphe Silva de Castro, Tamiris Bezerra Costa, Érika Patrícia Chagas Gomes Luz, Rodrigo Silveira Vieira, Igor Iuco Castro-Silva

**Affiliations:** 1 Universidade Federal do Ceará – Postgraduate Program in Dentistry – Fortaleza (CE) – Brazil.; 2 Universidade Federal do Ceará – Dental School – Sobral (CE) – Brazil.; 3 Universidade Federal do Ceará – Postgraduate Program in Chemical Engineering – Fortaleza (CE) – Brazil.

**Keywords:** Cellulose, Strontium, Hydroxyapatites, Materials Testing, Biocompatible Materials, Guided Tissue Regeneration

## Abstract

**Purpose:**

To evaluate membranes originating from pure or oxidized bacterial cellulose (BC)/alginate/strontium apatite hydrogels regarding toxicity, biocompatibility, biodegradation and metabolism.

**Methods:**

The toxicity was measured by incubating the materials with *Artemia salina* for 24 h, and mortality and the 50% lethal concentration were determined in comparison to potassium dichromate by Probit analysis. Local biocompatibility and biodegradation were evaluated by subcutaneous assay in 75 Swiss mice; the test groups were compared to sham and collagen membrane at one, three and nine weeks. The histopathology of tissue irritation followed the ISO 10993-6 standard, and the integrity of the biomaterials scored by quartiles. Metabolic analysis of relative weight and the intensity of catalase, iodine and nitrite were carried out for liver, kidneys and tibias of the tested animals.

**Results:**

All cellulose-based materials were nontoxic, biocompatible, and none presented nitrosative stress. The oxidized BC was more resorbable, and the non-oxidized BC had greater renal biochemical reactivity.

**Conclusion:**

The membranes suggest applicability as regenerative barriers. However, long-term studies in bone defects are necessary to elucidate their osteopromoting efficiency.

## Introduction

Severe orofacial bone loss of congenital, infectious, traumatic or neoplastic etiology negatively impacts quality of life and continues to be a challenge for full rehabilitation^1^. Over the last two decades, research on bone regeneration has evolved, with the development of a wide variety of implantable biomaterials and surgical protocols^2^^,^ 3. Barrier membranes are used for guided tissue regeneration of small periodontal defects, avoiding invasion of adjacent mucosa and maintaining a stable blood clot to favor osteogenesis, or in guided bone regeneration (GBR) in large alveolar ridge defects, maintaining a synergistic combination with mineral bone graft^4^. As desirable characteristics, regenerative membranes must present good physical properties, be easy to handle, model over the bone defect, have cellular occlusivity, maintain the intraosseous space, be biocompatible and undergo balanced degradation with the formation of new tissue^4^^–^ 6. Even under adverse conditions such as inflamed tissues, occlusive membranes have demonstrated a fundamental role in GBR and its resistance to degradation^6^. Due to biotechnological progress, there is the increasing and versatile development of different formats and compositions of implantable membranes^1^^,^ 5. Among them, polymer-polymer associations, to delay biodegradation, or polymer-ceramics, to combine the synergistic effects of resistance and hardness, mimicking the composite bone structure itself, based on collagen and apatite^1^^,^ 2.

Natural biopolymers that replace the classic collagen standard have attracted the production chain due to their sustainability and lower production costs, as it is the case with bacterial cellulose^7^. Bacterial cellulose is a linear polymer, derived from the fermentation of several non-pathogenic bacteria, especially those of the genus Komagataeibacter, and has the properties of good mechanical resistance, chemical stability, porosity, crystallinity, high hydrophilicity, purity and biocompatibility, being attractive for tissue engineering^7^^–^ 13.

The major limitation as a material for bone regeneration is its limited biodegradation due to its high polymer crystallinity and compact fibrous microstructure^7^^,^ 8 ^,^ 11. Oxidation is a chemical modification technique that can occur totally or partially, to modify some properties of the polymer^1^^–^ 5 and make it susceptible to degradation^8^^,^ 11. The association with apatite seeks to make cellulosic scaffolds biomimetic, and their implantation can promote bone formation^8^^–^ 12. Furthermore, apatites incorporated with the metal strontium in their structure can enhance their activity in the bone remodeling process, inhibiting reabsorption and stimulating new bone formation, without toxicity and favoring osteopromotion^8^^,^ 10 ^–^ 12.

In biomaterials research, the following criteria are used to analyze the product: synthesis, structure, and properties, balancing product design with physicochemical, mechanical and biological performance, which makes animal research extremely relevant and belonging to the base of the evidence pyramid^14^. Preclinical tests of biomaterials are necessary to validate biological safety, in recognition of their non-toxicity and biocompatibility, and efficacy, by generating some direct or indirect tissue stimulus together with the integrity compatible with the need and time of tissue regeneration^5^^,^ 11 ^,^ 15.

Thus, this study evaluated the acute toxicity, biocompatibility, biodegradation and *in-vivo* metabolism of hybrid membranes of pure or oxidized bacterial cellulose associated with sodium alginate and strontium apatite.

## Methods

### Ethical aspects

**Figure 1:**
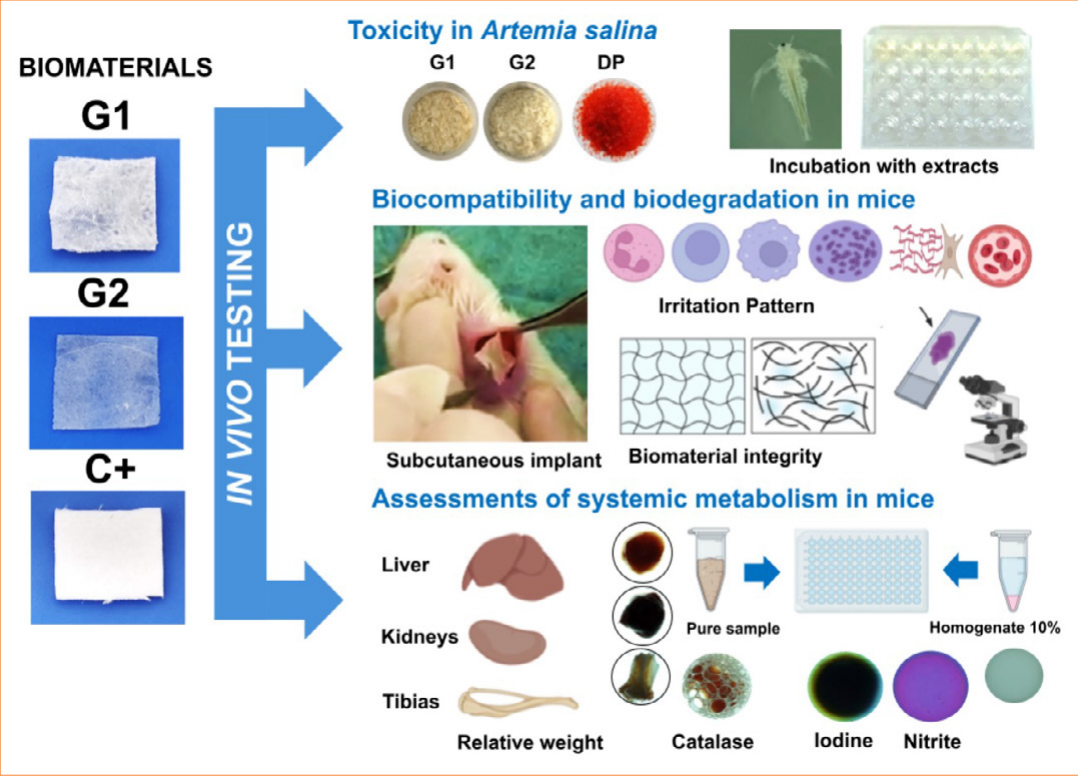
Experimental design of in-vivo tests for cellulose/alginate/strontium apatite membranes and controls.

Source: Elaborated by the authors.

This study adopted the international principles of Replacement, Reduction and Refinement in Animal Research: Reporting of *In Vivo* Experiments (3R-ARRIVE 2.0 guide)^16^. The experimental protocol was approved by the Animal Use Ethics Committee of the Universidade Federal do Ceará, Sobral, CE, Brazil, under registration number 02/2023. The synthesis of the *in-vivo* tests performed in this research is summarized in Fig. 1.

### Experimental groups

Two prototypes were tested in the form of membranes, according to their composition of hybrid hydrogels: pure bacterial cellulose (27%), sodium alginate (5%), strontium apatite (17%) and water (51%) (G1) or oxidized bacterial cellulose (39.1%), sodium alginate (4.2%), strontium apatite (14.2%) and water (42.5%) (G2). The membranes were produced by biotechnological and biomimetic processes, according to preliminary studies^8^^–^ 11, and previously freeze-dried and sterilized. A natural Lumina Coat bovine type I collagen membrane (Criteria, SP, Brazil), in the commercial dimension of 30 × 20 × 1 mm, resorbable in eight weeks according to the supplier’s information^12^, constituted the commercial control (C+). For testing in the different experimental models, all the membranes were handled aseptically.

### Acute toxicity test

The experimental model used was the microcrustacean *Artemia salina*, a bioindicator of acute toxicity according to ISO/TS 20787^17^. Microcrustacean cysts were incubated in saline water with alkaline adjustment (pH = 8) under constant aeration at 25°C for 48 h. After hatching, young larval forms (nauplii), mobile and phototropic, were collected with a pipette and plated in 24-well plates in quadruplicate at the density of 10 nauplii per 1 mL/well. Aqueous extracts of G1, G2 and C+ obtained after fragmentation of the test samples and immersion for 1 h in saline water were configured at concentrations of 10, 100, 1,000 and 10,000 μg/mL, and compared to the positive control (C+), potassium dichromate/PD (Êxodo Científica, SP, Brazil) applied at the same concentrations. The dead and total animals were counted after 24 h using an XTB-1B stereomicroscope (Coleman, Brazil), and the respective percentage of artemia mortality was calculated according to the Abbott formula defined in Eq. 1:


$$\%M_{x}=\left(M_{x}-M_{c.}/100-M_{c}\right)\times 100$$ (1)

where: % *M_x_*: corrected percentage of artemia mortality per test group or positive control; *M_x_*: percentage of artemia mortality per test group or positive control; *M_C-_*: percentage of artemia mortality in negative control.

The immobile nauplii were considered dead, and the descriptive analysis used the mean ± mean deviation per group. To estimate the representative value of the lethal concentration for 50% of the artemia (LC_50_), the Probit method was applied, using the linear regression equation defined in Eq. 2:


$$y=\beta_{o}+\beta_{1}\cdot x$$ (2)

where: y: dependent variable or mortality, considered 50 to determine the LC_50_; *β*_0_: intercept of the regression equation, calculated by Y_mean_ - (*β*_1_ x X_mean_); *β*_1_: slope of the regression equation, calculated by ∑ (X–X_mean_) (Y–Y_mean_) / ∑ (X–X_mean_)^2^; *x* = independent variable or concentration.

The threshold for the presence of toxicity was adopted as a LC_50_ in concentrations < 1,000 μg/mL.

### Ectopic subcutaneous implantation

The experimental model used was the heterogenic albino Swiss mouse (*Mus musculus*), male, young adult, with an average weight of 30 g. During the experimental period, the animals remained in collective cages according to the experimental group, in a climate-controlled vivarium, with a 12-hour light/dark cycle, pelleted food and water *ad libitum*. Sixty animals were distributed according to different experimental conditions (four groups, three times, five specimens each). Intraperitoneal anesthesia was administered with a solution of 10% ketamine (Dopalen, Sespo Indústria e Comércio Ltda, SP, Brazil) at the dose of 80 mg/kg, and 2% xylazine (Anasedan, Sespo Indústria e Comércio, SP, Brazil) at the dose of 10 mg/kg. A trichotomy of the trunk-dorsal region was performed, and antisepsis was performed with 0.5% aqueous chlorhexidine. A 1-cm linear incision was made, followed by tissue divulsion to form a subdermal pocket. Each animal received a subcutaneous implant corresponding to G1, G2 or C+ with a standardized dimension of 10 mm^2^ or remained without implant, only with the surgical bed filled with blood clot (C-). Then, the operated regions received simple sutures with mononylon 4.0 thread (Procare, Medico Industries & Trade Co, Shijiazhuang, China). In all groups and experimental times, the presence of postoperative infection was not observed.

At one, three and nine weeks after surgery, the animals were euthanized by an overdose of the anesthetic solution (three times the usual dose), and an immediate excisional necropsy of the skin area deepened at the level of the subcutaneous procedure was performed. In addition, liver, kidneys and tibias were also removed for metabolic studies.

All samples from the subcutaneous bed were fixed in 10% (v/v) buffered formalin solution, pH 7.0, for 48 h. After fixation, the necropsies were washed in running water for 1 h, cleaved longitudinally, dehydrated in increasing baths of 70–100% ethanol, bathed in xylene, impregnated and embedded in paraffin. The paraffin blocks were cut in 4-µm sections and stained with hematoxylin-eosin (HE). All histological slides were examined under the supervision of an experienced pathologist.

To characterize biocompatibility and biodegradation, a qualitative and quantitative analysis was conducted. Five images of each sample were captured in adjacent, non-overlapping fields in the center of the implant or surgically manipulated area along its entire length, using an Eclipse Ei microscope (Nikon, Tokyo, Japan) and a Prime Cam 12MP Pro digital capture system (Prime Life Science, Basel, Switzerland), with a total magnification of 1,600×. For qualitative analysis, slides from each experimental group were selected and morphologically described to represent the observed events. Quantitative analysis of biocompatibility or irritation pattern adopted the guidelines of ISO 10993-6^18^, considering the presence of neutrophils, lymphocytes, macrophages and foreign body giant cells as inflammatory criteria, while the presence of neovascularization and connective tissue as reparative criteria. The standard assesses the presence of such criteria by scores defined as 0 (absent), 1 (rare), 2 (moderate), 3 (intense) or 4 (overcrowding), generating a numerical system capable of determining the irritation pattern. To determine the irritation pattern of each condition (25 results, considering quintuplicates of animals and images of each test and control group), three equations were used: Eq. 3 or equation for the inflammation pattern (*I_x_*); Eq. 4 or equation for repair pattern (*R_x_*); and Eq. 5 or equation for irritation pattern:


$$I_{x}=2\Sigma (Nt+L+M+FBGC)$$ (3)

where: *I_x_*: inflammation pattern by test or control group; *Nt*: mean neutrophil score; *L*: mean lymphocyte score; *M*: mean macrophage score; *FBGC*: mean foreign body giant cell score.


$$R_{x}=\Sigma (Nv+CT)$$ (4)

where: *R_x_*: repair pattern by test or control group; *Nv*: mean neovascularization score; *CT*: mean connective tissue score.


$$IP_{x}=\left(I_{x}+R_{x}\right)-\left(I_{c-}+R_{c-}\right)$$ (5)

where: *IP_x_*: total irritation pattern by test group or positive control; *I_x_*: mean inflammation pattern score by group; *R_x_*: average repair pattern score by group; *I_C-_*: mean score of inflammation pattern in negative control; *R_C-_*: average score of the repair pattern in the negative control.

After the general calculations, the ISO 10993-6 standard adopts the negative control as the standard for the other groups, by subtracting it from the others, to reduce interpretation bias. Finally, the actual irritation pattern of the experimental groups corresponds to one of the following ranges: non-irritating (0.0–2.9), mildly irritating (3.0–8.9), moderately irritating (9.0–15.0) or severely irritating (> 15); the negative result is considered standard 0.

To histologically analyze biodegradation in the photomicrographs obtained in each test and control group, observing the guidelines of ISO 22803^19^ for regenerative membranes, a standard analogous to scores from 0 to 4 was proposed in parallel to grade the integrity or presence of the biomaterial by quartiles, defined as 0 (absent), 1 (minimal: up to 25%), 2 (mild: up to 50%), 3 (moderate: up to 75%) or 4 (predominant: above 75%).

The raw data were tabulated in Excel (Microsoft Office, United States of America), expressed graphically as mean ± standard deviation and statistically analyzed using Jamovi software version 2.3.28 (Jamovi Project, Australia) for comparisons between groups according to the criteria and experimental times described.

### Metabolic tests for chronic systemic effects

To investigate the potential chronic systemic impact of the implanted materials, kidneys and liver were chosen as classic general pathways of metabolic biotransformation, and tibias, as the largest long bones of the mouse to represent bone metabolism. A parallel pathological analysis of the key organs of all groups included records of the appearance of the anatomical specimens with the naked eye, including color, consistency, texture and relative weight (%) per animal and functional studies through biochemical analyses.

Fresh samples were kept frozen at -20°C until the time of the experiments, when they were used at room temperature. Three quantitative analytical techniques were performed:

- Iodine staining to identify possible residues of long-chain unbranched polysaccharides, such as cellulose;
- Catalase test to identify the preservation of the antioxidant reaction of native tissue to hydrogen peroxide;
- Identification of the presence of nitrite, a potential chronic cellular stressor.

For iodine staining, each sample was macerated in a mortar to obtain 10% homogenates in 50 mM phosphate buffer solution (pH 7.4), which remained in 1-mL Eppendorf microtubes, refrigerated at 4°C for 24 h. An aliquot of 180 µL of the supernatant of each homogenate was placed in an individual well of a 96-well plate, 20 µL of 2% Lugol’s strong solution (2% iodine and 4% potassium iodide, PROC9 Indústria Química, RS, Brazil) was dripped, and the reaction was observed for 1 minute at room temperature. The results were categorized, according to the change in hue of the iodine added to the samples, as 0 (absent or amber, using distilled water as the blank or negative control), 1 (weak or medium brown, using natural yogurt made from whole milk and yeast as the standard, Betânia, PE, Brazil), 2 (moderate or dark brown, using a mixture of starch and yogurt, 1:3 as the standard) and 3 (intense or bluish-black, using Maizena cornstarch as the standard, Duryea, SP, Brazil).

For the catalase test, each 4-mm diameter and 1-mm thick fragment obtained by punch and scalpel was placed in an individual well of a 96-well plate, 20 µL of 3% hydrogen peroxide solution (Millipore Sigma, United States of America) was dripped, and the effect of active endogenous catalase, dissociating hydrogen peroxide into oxygen gas and water, was observed at room temperature for 15 seconds (for kidney and liver) and 30 seconds (for bone). The results were categorized according to the intensity of bubbling in the samples studied as 0 (absent, using distilled water as blank or negative control), 1 (weak, using kidney as standard), 2 (moderate, using bone as standard), and 3 (intense, using liver as standard).

For nitrite identification, each sample was macerated in a mortar to obtain 10% homogenates in 50 mM phosphate buffer solution (pH 7.4), which remained in 1-mL Eppendorf microtubes, refrigerated at 4°C for 24 h. An aliquot of 188 µL of the supernatant of each homogenate was placed in an individual well of a 96-well plate, mixed with 6 µL of reagent 1 (sulfanilic acid, acetic acid and distilled water), then with 6 µL of reagent 2 (alpha-naphthylamine and ethyl alcohol) from the nitrite detection kit (Nitrite NO_2_-, LabconTest, SC, Brazil), reacting for 10 minutes at room temperature. In the Griess-Ilosvay method, nitrite reacts with reagent 1, forming a diazo compound that interacts with reagent 2, giving the solution an intense pink color. The reactions of the samples were categorized according to comparative colorimetry to the indicators of the commercial kit as 0 (ideal or 0 ppm, using distilled water as blank or negative control), 1 (acceptable or 0.25 ppm, using as standard ham, Seara, SC, Brazil), 2 (critical or 0.5 ppm, using as standard hotdog sausage, Perdigão, SC, Brazil) and 3 (dangerous or ≥ 1 ppm, using as standard cooked and smoked mixed sausage, Seara, SC, Brazil). All tests were interpreted by two examiners in agreement and supervised by an experienced pathologist.

### Statistical analysis

The data distribution was subjected to the Shapiro-Wilk’s test, and, once its non-normality was confirmed, the non-parametric Kruskal-Wallis’s test and the Dwass-Steel-Critchlow-Fligner’s multiple comparisons post hoc test were applied, considering a 95% confidence interval and significant differences if *p* < 0.05.

## Results

### Acute toxicity analysis

**Figure 2:**
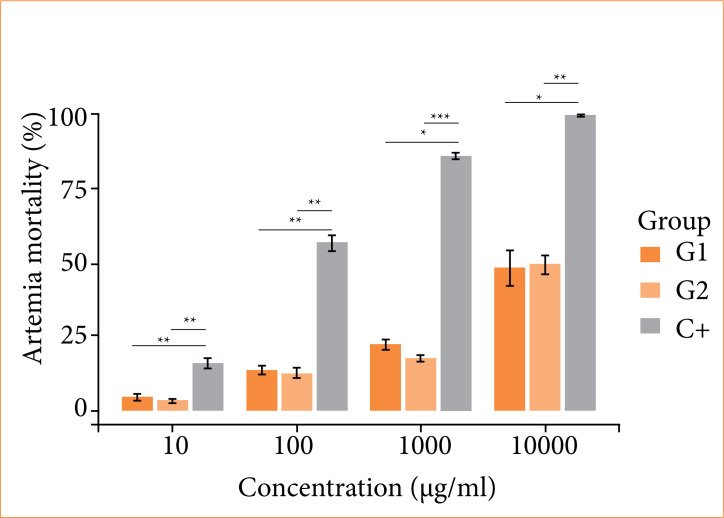
Acute toxicity assay for cellulose/alginate/strontium apatite membranes and control in artemia nauplii after 24 h of exposure.

Concentration-dependent mortality was observed for all groups. G1 and G2 had no statistical differences between them but had mortality significantly lower than C+, notably toxic at low concentration, thus indicating the absence of membrane toxicity in the microcrustacean model (Fig. 2).

*p = 0.01–0.04, **p = 0.001–0.009; ***p < 0.001. Source: Elaborated by the authors.

In addition, linear regression analysis from the mortalities obtained at the tested concentrations identified a very low LC_50_ for C+ (LC_50_ = 45.80, Y = 49.75730564 + 0.005299124883X) compared to the limit of 1,000 µg/mL, which represents evident toxicity. The test groups had estimated scores higher than 10,000 µg/mL and like each other, when comparing G1 (LC_50_ = 10.366.85, Y = 11.91239958 + 0.003673978549X) and G2 (LC_50_ = 10.112.69, Y = 9.576139456 + 0.00399733953X), corroborating the initial biological safety of the membranes.

### Analysis of in-vivo biocompatibility

**Figure 3:**
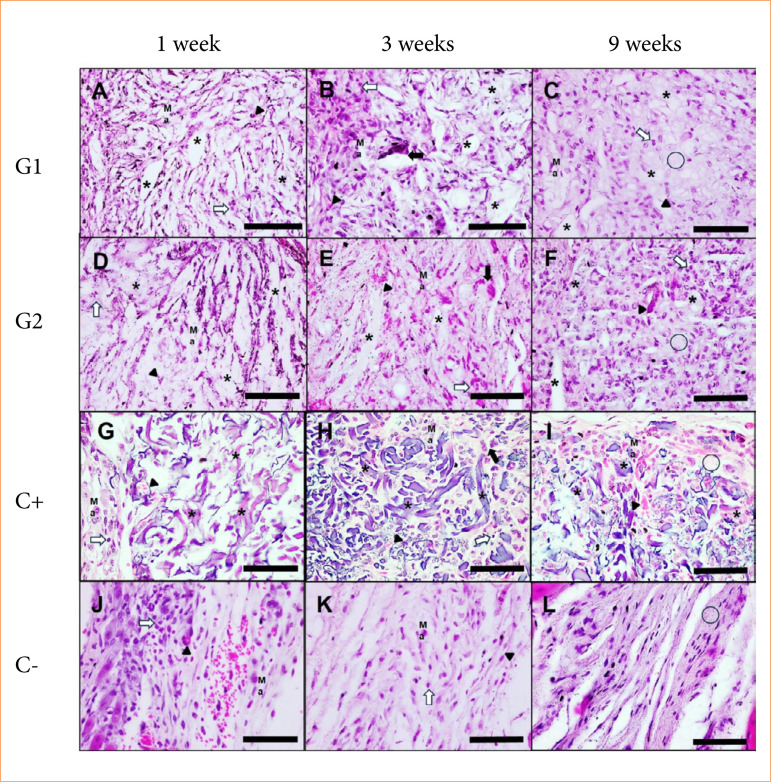
Histological analysis of biocompatibility and biodegradation of cellulose/alginate/strontium apatite membranes and controls implanted subcutaneously in mice after one, three and nine weeks.

The descriptive histological analysis showed a gradual decrease in the inflammatory infiltrate and replacement by fibroblast-like cells and collagenous matrix for C-. For G1 and G2, there was a clear increase in cellularity from one to three weeks and a small improvement in matrix repair from three to nine weeks. The presence of filamentous, multilaminar, thick and multidirectional fragments, unstained or slightly eosinophilic, compatible with the polysaccharide composition of bacterial cellulose and the adsorption of proteins to apatite in G1 and G2, was reduced after three weeks, and there was replacement by granulation tissue. C+ showed greater cellularity in the form of granulation tissue and a decrease in filamentous, thin, multidirectional and basophilic fragments compatible with xenobiotic collagen at nine weeks. Neither group exhibited a dense fibrous capsule, characteristic of a response to a foreign body (Fig. 3).

*Implanted material; white arrow: mixed inflammatory infiltrate; black arrow: giant cell; arrowhead: blood vessel; circle: fibrosis. Staining: hematoxylin-eosin. Magnification: 1,600 x. Scale bar: 600 μm. Source: Elaborated by the authors.

**Figure 4:**
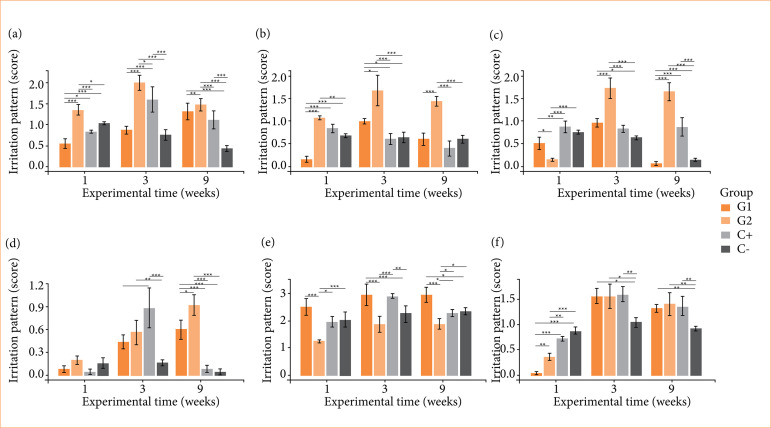
Intensity of biocompatibility criteria for cellulose/alginate/strontium apatite membranes and controls implanted subcutaneously in mice after one, three and nine weeks: **(a)** neutrophils, **(b)** lymphocytes, **(c)** macrophages, **(d)** giant cells, **(e)** fibrosis and **(f)** neovascularization.

Bacterial cellulose-based membranes exhibited heterogeneous responses in quantitative histological analysis in comparison with C-. The intensity of neutrophils was higher for G2 and lower for G1 at one week, higher for G2 and C+ at three weeks and higher for G2, G1 and C+ at nine weeks. The intensity of lymphocytes was lower for G1 at one week, higher for G2 at one and three weeks and higher for G2 at nine weeks. The intensity of macrophages was lower for G2 at one week, higher for G2 and G1 at three weeks and for G2 and C+ at nine weeks. The intensity of multinucleated giant cells of the foreign body type was low and did not vary at one week, increased and was higher for C+ and G2 at three weeks and for G2 and G1 at nine weeks. The intensity of fibrosis was lower for G2 at one week, higher for G1 and C+ at three weeks and higher for G1 and lower for G2 at nine weeks. The intensity of neovascularization was lower for G1 and G2 at one week, higher for C+, G1 and G2 at three weeks and higher for G2, C+ and G1 at nine weeks. These results showed a persistent neutrophilic, lymphocytic, macrophagic and multinuclear reactive pattern for G2 and a more fibrogenic profile for G1 (Fig. 4).

The general irritation pattern of hydrogels G1 and G2 ranged from non-irritating to mildly irritating considering one and nine weeks respectively, while C+ remained as non-irritating. An increase in irritation was noted throughout the experiment for all groups, with hydrogels G1 and G2 exhibiting the greatest change from one to three weeks and peak at nine weeks, and C+ exhibiting the greatest change and peaking at three weeks and decrease at nine weeks (Table 1).

*p = 0.01–0.04; **p = 0.001–0.009; ***p < 0.001. Source: Elaborated by the authors.

**Table 1:** Irritation pattern of cellulose-based hybrid membranes and commercial control in subcutaneous tissue in mice at one, three and nine weeks.

Experimental times (weeks)	Experimental groups		
	G1	G2	C+
One	0.00 (NI)	0.00 (NI)	0.00 (NI)
Three	3.40 (MI)	7.68 (MI)	4.68 (MI)
Nine	3.72 (MI)	8.48 (MI)	2.84 (NI)

G1: pure bacterial cellulose/alginate/strontium apatite; G2: oxidized bacterial cellulose/alginate/strontium apatite; C+: collagen; NI: non-irritating; MI: mildly irritating. Source: Elaborated by the authors.

### Analysis of in-vivo biodegradation

**Figure 5:**
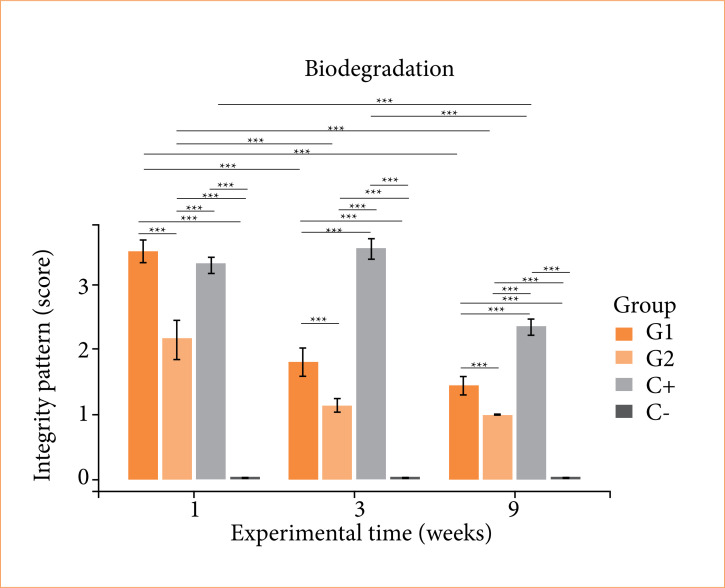
Integrity of cellulose/alginate/strontium apatite membranes and controls implanted subcutaneously in mice after one, three and nine weeks.

The integrity of the biomaterials showed intragroup differences, with G1 and G2 showing a decrease at three weeks and a plateau at nine weeks, and C+ showing a decrease only at nine weeks. In the intergroup comparison, there was a greater presence of fragments at one week for G1 and C+ compared to G2, while at three and nine weeks, for C+ compared to G1 and G2. This indicates lower biodegradability in C+, intermediate in G1 and high in G2 (Fig. 5).

*p = 0.01–0.04; **p = 0.001–0.009; ***p < 0.001. Source: Elaborated by the authors.

### Analysis of in-vivo systemic effects

The key organs of all groups did not show differences in the normotypical macroscopic appearance in reference to C-, with the liver samples remaining brown in color, fibrous consistency and smooth texture, kidneys in dark-brown color, fibrous consistency and smooth texture and tibias in light color, hard consistency and smooth texture.

**Figure 6:**
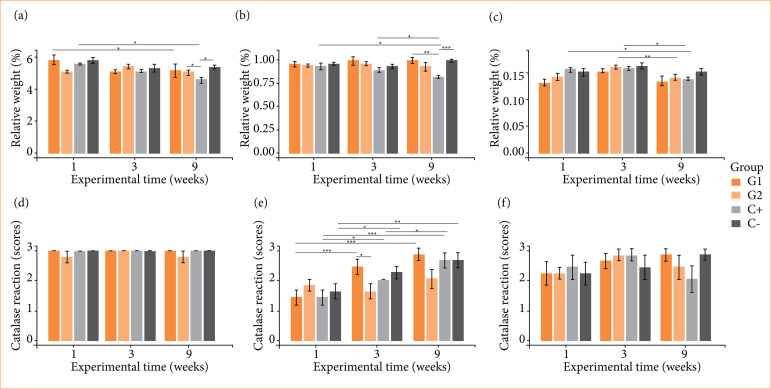
Metabolism tests of cellulose/alginate/strontium apatite membranes and controls implanted subcutaneously in mice after one, three and nine weeks: relative weight for **(a)** liver, **(b)** kidneys and **(c)** tibias and catalase reaction for **(d)** liver, **(e)** kidneys and **(f)** tibias.

In the analysis of relative weight for liver, there were intergroup differences by experimental time compared to C-, with C+ having the lowest relative weight at nine weeks. Intragroup differences were identified from one to nine weeks, with a decrease in relative weight for C+ and G1 (Fig. 6a). For kidneys, there were intergroup differences by experimental time compared to C-, with C+ exhibiting a lower relative weight at nine weeks. Intragroup differences were identified from one and three weeks to nine weeks, with a decrease in relative weight for C+ (Fig. 6b). For tibias, there were no intergroup differences by experimental time, but intragroup differences were identified, from one to nine weeks with a decrease in relative weight for C+ and from three to nine weeks, with a decrease in weight for G2 and C+ (Fig. 6c). The endogenous catalase reaction was greater and very homogeneous for liver compared to the other organs, with no differences between groups or experimental times (Fig. 6d). For kidneys, there were no intergroup differences by experimental time compared to C-, but intragroup differences were identified, from one to three and nine weeks with an increase in the catalase reaction for G1, C+ and C- (Fig. 6e). For tibias, there were no intergroup or intragroup differences (Fig. 6f).

*p = 0.01–0.04; **p = 0.001–0.009; ***p < 0.001. Source: Elaborated by the authors.

**Figure 7:**
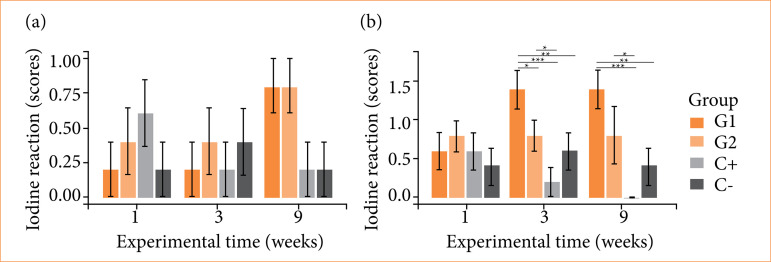
Metabolism tests of cellulose/alginate/strontium apatite membranes and controls implanted subcutaneously in mice after one, three and nine weeks: iodine for **(a)** liver and **(b)** kidneys. Iodine reaction for tibias and nitrite reaction for liver, kidneys and tibias had null reaction (data not shown).

The reaction to iodine for liver did not show intergroup or intragroup differences (Fig. 7a). For kidneys, there were intergroup differences by experimental time compared to C-, at three and nine weeks with G1 showing a greater reaction to iodine, with no intragroup differences (Fig. 7b). For tibias, the reaction was null for all groups, with graphical data not shown.

Unlike previous reactivities in key organs, the reaction to nitrite followed a nonspecific null pattern for all organic tissue samples, groups and experimental times analyzed, with graphical data not shown.

*p = 0.01–0.04; **p = 0.001–0.009; ***p < 0.001. Source: Elaborated by the authors.

## Discussion

The promising performance of hydrogels in the *Artemia salina* model is reinforced by high LC_50_, well above the range of up to 1,000 µg/mL, observed in initial screening in nauplii of various plant extracts with toxicity^20^. In a preliminary test of a hybrid hydrogel of collagen gelatin/methacrylate and laponite silicate for bone application using the artemia model at 24 and 48 h, there was no significant acute toxicity, although the group without the mineral in the highest concentration (5 mg/mL) reached 60% viability, a margin still higher than LC_50_ 21, showing similarity of this composite to hybrid cellulosic biomaterials.

Hybrid natural biomaterials are potential candidates for regenerative membranes, but they do not always yield synergistically more favorable biological results in the subcutaneous bed of mice. A poultry composite of 90% collagen: 5% nanokeratin: 5% apatite generated greater irritation, from 1.56 (non-irritating) in one week to 3.00 (mildly irritating) in nine weeks, compared to pure collagen, 90% collagen: 10% nanokeratin or 90% collagen: 10% apatite, groups in the range of 0.0 to 1.64 in one week and 0.00 to 1.88 in nine weeks, the last one considered non-irritating^22^. Fish-derived collagen-apatite composites exhibited decreasing irritation over time and increasing irritation with higher mineral content, ranging from 6.64 (mildly irritating) to 9.12 (moderately irritating) in one week and from 2.60 (non-irritating) to 4.08 (mildly irritating) in nine weeks, in compositions of 80% collagen:20% apatite to 60% collagen:40% apatite^23^. Despite increasing irritation from one to nine weeks and heterogeneous cellular or tissue responses, the different hybrid cellulosic biomaterials tested were in the mildly irritant range, like most hybrid collagenous biomaterials, which make them biocompatible according to international standards^18^.

The bacterial cellulose-based membranes derived from hybrid hydrogels were designed to more closely match the bone composition, presenting a higher strontium apatite ratio than other studies, in which composites with a predominance of bacterial cellulose exhibited evident biocompatibility, but more limited biodegradation in follow-ups for up to nine weeks in subcutaneous implants in mice^8^ or up to three months in rat calvaria^11^. On the contrary, the use of oxidized bacterial cellulose already with expected biodegradation compared to the pure form^8^^,^ 11, in our study with a proportion greater than twice that of strontium apatite, would seek to limit the bias of very rapid reabsorption and achieve its functionality as a barrier to membrane for bone applications^4^^–^ 6. Tissue irritation of hybrid membranes made of pure or oxidized bacterial cellulose, sodium alginate and strontium apatite is anchored in the *in-vivo* biological responses expected in the literature for biomaterials with a polymeric/ceramic structure, converging with an expected and progressive decrease of neutrophils, lymphocytes and macrophages and a moderate increase in giant cells, fibrosis and neovascularization throughout the experimental weeks^8^^,^ 11 ^,^ 24.

Neutrophils represent acute inflammation occurring in the period of three to 10 days, responding nonspecifically to polymeric or ceramic implants, natural or synthetic, acting as phagocytes of the host’s innate immunity^24^. Its persistence beyond this period would indicate an acute condition that deserves to be investigated, since the large generation of reactive oxygen species in the implanted biomaterial can cause tissue damage and culminate in necrosis in more severe cases^24^^,^ 25. Neutrophil concentrations vary according to membrane composition, with a tendency to decrease: high to low from one to 12 weeks with polyglycolate (PLGA) and poly(trimethylene carbonate) (PTMC)^26^, high to low one to nine weeks with collagen-apatite^23^, moderate to low one to nine weeks with collagen-apatite-nanokeratin^22^ or moderate from one to three weeks and absent in nine weeks with alginate-capsul^27^.

Lymphocytes represent chronic inflammation, normally detectable from three days, with an intensified presence from 10 to 30 days if the biomaterial is resorbable, such as collagen membranes, or of synthetic origin, such as mineral grafts, acting as signaling cells of the inflammatory process^24^. Lymphocyte concentrations vary according to membrane composition, with a decreasing trend: high to low from one to 12 weeks with PLGA and PTMC^26^, moderate to low one to nine weeks with collagen-apatite^23^, low up to three weeks and zero in nine weeks with collagen-apatite-nanokeratin^22^ or moderate from one to three weeks and low in nine weeks with alginate-capsul^27^.

Macrophages are phagocytic cells detectable from three days onwards, increasing between 10 and 30 days and decreasing in the case of non-resorbable materials, but resorbable collagen scaffolds have persistent and more intense activity up to 60 days, while granular mineral bone grafts exhibit a moderate presence between 10 and 60 days, regardless of the natural or synthetic origin of the biomaterial^24^. Macrophage concentrations vary according to membrane composition, with a decreasing tendency: high to moderate from one to 12 weeks with PLGA and PTMC^26^, low from one to nine weeks with collagen-apatite^23^ and low from one to nine weeks with collagen-apatite-nanokeratin^22^. Furthermore, irregular and large hydroxyapatite microparticles induced reactivity in macrophages, generating high levels of interleukin (IL)-1β through activation of the inflammasome pathway^28^.

Giant cells are rare in non-resorbable biomaterials and play a prominent role in mediating the reabsorption of collagen membranes, appearing between 15 and 30 days but intensifying their presence and catalytic activity of the implant from 30 to 60 days, after which they decrease^24^. In mineral granular materials, they appear a little earlier, between 10 and 15 days, and are exacerbated from 30 to 60 days in those of synthetic origin, while in natural grafts they tend to regress in the same period^24^. Giant cell concentrations vary according to membrane composition, with a tendency to increase followed by decline: zero in one week, low in three weeks and moderate in nine weeks with alginate-capsul^27^, zero at one week and high to moderate at three to 12 weeks with PLGA and PTMC^26^, low in one week, moderate in three weeks and low in nine weeks with collagen-apatite^23^ and zero in one week to low in three to nine weeks with collagen-apatite-nanokeratin^22^.

The pathognomonic feature of the foreign body response to an implanted material is encapsulation by very dense fibrous tissue^14^^,^ 29. However, the permeation of loose connective tissue is a natural event in the healing process, with invasion of fibroblasts into the spaces between fragments and sometimes adherent to the biomaterial, more evident after 10–15 days, with collagen fibrillogenesis up to 30 days and more intense fibrogenesis up to 60 days^24^. The acceleration of the maturity of the host collagen matrix represents an important subject of analysis, since, while a viscoelastic extracellular matrix favors cell adhesion, proliferation and differentiation^30^, the presence of intense fibrosis restricts a desirable osteogenesis^12^. Fibrosis or connective tissue concentrations vary according to membrane composition, with a tendency to increase: moderate to high from one to three weeks with collagen-apatite^23^, low to moderate from one to nine weeks with collagen-apatite-nanokeratin^22^, zero in one week and low in three and nine weeks with alginate-capsul^27^. The implanted cellulosic materials were beneficial because they did not present a foreign body or fibrous capsule type response, with the non-oxidized form being more susceptible to fibrogenesis than the oxidized form. This explanation could reside in the selective crosstalk between M1/M2 macrophage subtypes with fibroblasts, in which an M1 lineage present up to three days post-injury if overstimulated would induce chronicity of the inflammatory phenomenon, while M2 present up to one week if overstimulated would induce greater potency of anti-inflammatory or fibrogenic response to implanted biomaterials^29^.

Neovascularization in implanted biomaterials is discreet and begins at the periphery, with invasion of small-caliber vessels between 10 and 30 days and remaining stable in granular mineral grafts, while in resorbable collagen matrices there is a tendency for an increase from 30 to 60 days, which favors the later transit of phagocytes involved in the disintegration of the biomaterial^24^. The degree of vascularization is one of the important parameters for evaluating the potential of biomaterials, since a microenvironment that allows satisfactory nutrition, adhesion and differentiation of osteogenic cells is favorable for GBR, with small pores or interconnections of 100–150 µm being more vasculogenic while those above 400 µm are more fibrogenic^31^. Neovascularization concentrations vary according to membrane composition, with a tendency towards stability: stable from high to moderate from one to 12 weeks with PLGA and PTMC^26^, stable moderate from one to nine weeks with collagen-apatite^23^ and increasing from low to moderate from one to nine weeks with collagen-apatite-nanokeratin^22^.

Unlike non-resorbable collagen fibrillar scaffolds up to 60 days, resorbable biomaterials undergo intense disintegration from 30 to 60 days, while synthetic mineral bone substitutes exhibit a decrease in the presence of granules and the size of their fragments between 30 and 60 days, more evidently than xenogeneic natural materials^24^. Synthetic polymeric blend of alginate-capsule exhibited resorption only in nine weeks, with residues smaller than 100 µm, evoking a moderate and continuous inflammatory response from one to nine weeks^27^. Experimental composites of collagen and apatite at higher concentrations, from 40 to 60% mineral^23^, exhibited greater integrity than those with a content of 5 to 10%^22^, but there was no improvement in the performance of commercial non-crosslinked pure collagen up to nine weeks *in vivo* 23, which is like that observed in the cellulosic groups with strontium apatite in relation to the positive control. In the comparison of hemostatic adhesives, those based on oxidized cellulose were degraded in 2 h in human plasma, had greater swelling and were completely replaced by granulation tissue in 14 days in rabbit liver microscopy, in relation to those based on collagen, mechanically stable up to 8 h, with a maintained structure and permeated with little cellular infiltration *in vivo* 32, showing that oxidized cellulose may have a faster degradation. Cellulose-strontium apatite scaffolds implanted in subcutaneous tissue of mice^8^ or in rat calvaria^11^ exhibited more evident signs of degradation for the oxidized form, in nine weeks and three months, respectively^8^^,^ 11. The association with bioactives can also modulate the biodegradation phenomenon, case of biomaterials with strontium bound to calcium phosphates, which inhibit osteoclastic differentiation and limit bone resorption^31^.

Our research demonstrated the opposite, due to the greater stimulation of local degradation of biomaterials with cellulose and strontium apatite, which could hypothetically be justified by the determining action of oxidized cellulose or even by a differential action of strontium in resorptive cells of the connective tissue itself, such as macrophages and giant cells. Furthermore, thickness and pore size can influence biodegradation, as observed in permeable synthetic polymer blends of PLGA and PTMC implanted in the subcutaneous tissue of mice, in which a 100-µm thick membrane had greater and earlier biodegradation compared to a 250-µm membrane, with partial presence of fragments up to 12 weeks^26^.

The weight of the biomaterials instigated an understimulatory effect on C+. Histological study of oxidized cellulose and porcine gelatin membranes showed similarity of both to control direct bleeding in liver lesions in rats and biocompatibility up to 14 days, with chronic inflammation according to normal limits^33^. Another study with oxidized cellulose hemostatics in abdominal surgery in pigs demonstrated in six weeks that there were no differences in direct contact with the liver and spleen regarding inflammation, fibrosis, foreign bodies and hemorrhage^34^, thus refuting the role of oxidized cellulose in this explanation. Another microscopic study in the liver showed that the administration of strontium ranelate generated changes in the hepatic parenchyma of rats in 15 days, with sinusoidal dilation and more pronounced perisinusoidal fibrosis, while the control exhibited mixed inflammation and Kupffer cell hyperplasia^35^. The hepatic microsomal enzyme system may be responsible for the metabolization of a wide range of nonpolar or lipophilic products for elimination in the feces or even helping in the transformation into polar products for elimination in the kidneys^31^. Furthermore, glutathione-mediated biotransformation in the liver modulates nanoparticle transport^36^. In this sense, the increase in liver volume may be more likely associated with post-surgical recovery, constituting a reversible change in the liver parenchyma^35^, than a dysfunctional mechanism of hepatotoxicity^37^, since catalase activity remained stable.

Although studies claim that hydrophilic polymers injected subcutaneously in mice, such as polyethylene glycol, follow a distant distribution predominantly in the kidneys, but also in the heart, lungs and liver^38^, there was no notable change in renal weight. Instead, bone anabolism is promising and deserves to be investigated. A study of cellulose-strontium apatite implants in rat calvaria did not show significant bone formation *in loco* 11. Although the choice of key organs is sufficient to understand the primordial kinetics of the biomaterials in question, research in other organs is also encouraged.

Intracellular endogenous catalase is found in peroxisomes and in organs involved in the elimination of xenobiotics and may be increased in liver and kidneys exposed to strontium ranelate in ovariectomized rats, thus preventing oxidative stress^39^. The results of our study converge with oxidized bacterial cellulose presenting lower expression of catalase, but diverge from the hepatic site, indicating changes restricted to the kidneys, which may be contributory and unexplored data in the literature, suggesting biodistribution selectivity for cellulosic materials.

The tendency of iodine to increase in cellulose groups and decrease in collagen control may reside in the inherent organic properties of the polymers, of saccharide or protein order, respectively. The pharmacokinetics of polysaccharide residues for the kidneys, a well-vascularized organ permeable by fenestrated capillaries, is consistent with the biotransformation of hydrophilic compounds in the nephrons and elimination of these xenobiotics in the urine^40^. In this context, given the continuous blood distribution of such polysaccharide residues for up to nine weeks, future studies may delve deeper into the chemical process of glycation of proteins such as collagen, whose modification causes stiffening and loss of functionality, in addition to the generation of advanced glycosylation end products (AGEs) as occurs in hyperglycemic conditions, which increase the release of free radicals that cause oxidative damage^41^^,^ 42.

Nitrite constitutes an intermediate oxidation state of nitrogen, causing health concerns as it provokes methemoglobinemia, a disease that reduces the availability of oxygen to the body’s cells, and is involved in the formation of nitrosamines, compounds with carcinogenic potential^42^ with its maximum permitted value in drinking water equivalent to 1 mg/L^43^. This inorganic substance was not identified in the samples, thus ensuring systemic biological safety for local cellulosic implants.

## Conclusion

All bacterial cellulose-based materials were nontoxic, biocompatible, and none presented nitrosative stress. The oxidized bacterial cellulose was more resorbable, and the non-oxidized bacterial cellulose had greater renal biochemical reactivity. Future studies in bone defects are needed to elucidate the osteopromoting efficacy of the membranes as regenerative barriers.

## Data Availability

All datasets were generated or analyzed in the present study.
